# CLINICAL AND EPIDEMIOLOGICAL ANALYSIS OF SUICIDE ATTEMPTS IN CHILDREN ASSISTED BY A POISON CONTROL CENTER

**DOI:** 10.1590/1984-0462/2021/39/2019345

**Published:** 2020-10-28

**Authors:** Rafaele Maria Tirolla, Edmarlon Girotto, Camilo Molino Guidoni

**Affiliations:** a Universidade Estadual de Londrina, Londrina, PR, Brazil.

**Keywords:** Poisoning, Children, Suicide, attempted, Poison control centers, Intoxicação, Crianças, Tentativa de suicídio, Centros de Controle de Intoxicações

## Abstract

**Objective:**

To assess suicide attempts in children seeking care at a Poison Control Center.

**Methods:**

Cross-sectional study with children (<12 years old) that attempted suicide and were cared at the Poison Control Center in Londrina, Paraná, Southern Brazil, from April 1985 to December 2018.

**Results:**

We identified 59 children, most of them females (74.6%), who used only one product (77.9%). Among the products involved, medications were the most important ones (88.1%). Neurological/psychiatric/muscular manifestations (61.0%) were the main symptoms presented. The main reason identified for the suicide attempt was conflicts with family and/or friends (27.1%). Suicide attempts were more frequent in 2001-2003 and 2016-2018.

**Conclusions:**

Suicide attempts occurred mainly in female children with a single agent (mainly medications), and the main reason was family conflicts..

## RESUMO

**Objetivo:**

Analisar as tentativas de suicídio em crianças atendidas em um Centro de Informação e Assistência Toxicológica (CIATox-Londrina).

**Métodos:**

Estudo transversal com crianças (<12 anos) atendidas no CIATox-Londrina, Paraná, de abril/1985 a dezembro/2018, com tentativa de suicídio.

**Resultados:**

Identificaram-se 59 crianças, com predomínio do sexo feminino (74,6%) e com o uso de apenas um produto (77,9%). Entre os produtos envolvidos, destacaram-se os medicamentos (88,1%). As manifestações neurológicas/psíquicas/musculares (61,0%) foram os principais sintomas apresentados. O principal motivo identificado da tentativa de suicídio foram os conflitos com familiares e/ou amigos (27,1%). Houve maior frequência de tentativas de suicídio nos triênios 2001-2003 e 2016-2018.

**Conclusões:**

As tentativas de suicídio ocorreram predominantemente nas crianças do sexo feminino, com um único agente (em geral, medicamentos), em que o principal motivo foram os conflitos familiares, observando-se um aumento ao longo dos anos.

## INTRODUCTION

Social and psychosocial determinants are present in cases of toxicological events and, in this sense, suicide attempts stand out.^1^More than 800,000 people die from suicide each year globally, with suicide attempts being even more frequent. This is a global phenomenon and corresponds to an important public health problem, also affecting younger populations.^2^

Vulnerability to suicidal behavior in childhood, whose age corresponds to individuals up to 12 years old, is multifactorial. Among the main related factors are the incidence of mental disorders, bullying, school problems, exposure to violence, sexual abuse, family conflicts and the occurrence of suicide in the family.^3^ ^,^ ^4^ ^,^ ^5^

It appears that children do not usually expose this ideation verbally in comparison with other age groups, such as adolescents. Children can demonstrate a subtle change in behavior during this period, presenting it in a more withdrawn manner, which often does not strike parents or other guardians as odd. Thus, preventing suicide in children is difficult.^3^

From 2011 to 2016, there were 48,204 occurrences of suicide attempts in Brazil in the 10 to 19 year old age group,^6^with suicide being the 2nd leading cause of death in the 15-29 age group in 2016. In general, suicide is the 2nd leading cause of mortality among girls and the 3rd among boys. Hanging, the use of firearms and poisoning are methods commonly used in suicide attempts.^7^Hanging is the most used form by boys and drug poisoning, the use of sharp objects and firearms are preferably used by girls.^8^

In Brazil and in Paraná, from 2007 to 2017, there were, respectively, 14,584 and 2,350 suicide attempts, accounted for by toxicological events in individuals aged 10‒4 years.^9^ ^,^ ^10^Suicide or suicide attempts in childhood occur in smaller proportions, when compared with other age groups. However, they must be treated with relevance, since they constitute a tragic event and are increasing.^11^When analyzing epidemiological data on toxicological events in this age group, little significant figures are noted. However, it should be noted that data on attempted suicide are not regularly reported, and may not accurately express reality.^12^

In Brazil, there are few epidemiological studies related to suicide attempts in childhood. However, epidemiological data from other countries indicate an increase of this event in this age group.^8^Given the worldwide scenario and the scarcity of statistical data related to this event, especially in Brazil, this study aims to analyze suicide attempts in children treated at Poison Control Centers (CIATox).

## METHOD

This is a descriptive, cross-sectional study, conducted with all children (<12 years) seeking care at CIATox in Londrina (CIATox-Londrina). The Child and Adolescent Statute (ECA), Law No. 8,069, of 1990, considers as children all individuals aged up to 12 years old.^13^

CIATox-Londrina provides guidance and assistance in cases of accidents with venomous animals and intentional or accidental intoxications, as well as any contact with exogenous substances. It is located at the University Hospital of Universidade Estadual de Londrina (UEL), being a reference for the northern macro-region of the state of Paraná.

The study population was composed of all cases of suicide attempts in children registered by the service from April 1985 to December 2018. Until 2016, CIATox-Londrina used printed forms for notification and evolution of cases of intoxication and poisoning. As of 2018, the notifications made by CIATox-Londrina started to be registered in the Brazilian Intoxication Data System (DATATOX). Thus, all cases of the referred period were evaluated, analyzing the service's specific forms or data from DATATOX.

The study variables were collected from CIATox-Londrina databases, including age (in years), sex (female; male), year of occurrence (characterized in three-year periods - except 1985), time to initial care (1≤ hour; 1 to ≤2 hours; 2 to ≤24 hours;> 24 hours), health service responsible for initial care (hospital; emergency care unit; basic health unit), number of products used, classification of agent used (medicines; pesticides; household cleaning products; caustics; rodenticides), route of exposure (oral; others), gastric decontamination measures (emesis; gastric lavage; activated charcoal; gastric lavage + activated charcoal), use of antidotes/antagonists (yes; no), clinical manifestations (yes; no), reason and/or factors related to attempted suicide (conflicts with family/friends; psychiatric disorders; sexual abuse; bullying; other reasons), hospitalization (yes; no), length of hospital stay (in days), patient evolution (discharge; death).

The suicide attempt rate was calculated for each three-year period analyzed. For this calculation, the number of suicide attempts by children per three-year period was used as a numerator and the number of children who received care in the service per three-year period as a denominator, multiplying it by one thousand.

The data obtained were entered into a Microsoft Excel spreadsheet. Data processing and analysis was performed using the Statistical Package for the Social Sciences (SPSS), version 19.0, IBM Corp., Armonk, NY, USA. Descriptive statistical analysis was performed to examine the data, with simple frequency for qualitative variables and measures of central tendency for quantitative ones. The research was approved by the Research Ethics Committee of UEL (CAAE 45986415.1.0000.5231).

## RESULTS

**Figure 1:**
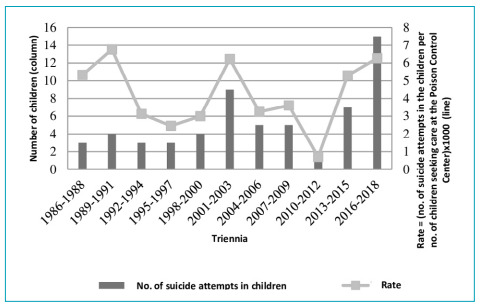
Suicide attempts in children in trienniums, CIATox-Londrina, April 1985 to December 2018.

In the period under study (April 1985 to December 2018), 59 children seen at CIATox-Londrina were identified for suicide attempts, with the highest number of cases taking place in the 2001-2003 and 2016-2018 trienniums. There is an increase in the number of absolute cases over the three-year periods, with an irregular distribution of the occurrence rate (Figure 1). Age ranged from 6 to 11 years, with a mean of 10.4±1.0 years, and the highest frequency was observed in females (74.6%; n=44).

The initial care of these children occurred between 15 minutes and 48 hours from the time of the toxicological event. However, in 11.9% of cases, the timing of the event was not identified. In 30 (50.8%) children, care took place from 2 to ≤4 hours (50.8%; n=30).

Hospitals stood out as the health service responsible for the initial care of these patients and for the request for care to CIATox-Londrina (83.0%; n=49). Of these children, 62.7% (n=37) were hospitalized (1-8 days), with an average of 1.7±1.8 days of hospitalization. None of the patients evolved to death.

In all cases, the route of exposure for suicide attempts was oral (100%; n=59). There was a variation of one to ten agents involved in each case of attempted suicide, and in most cases (78.0%), only one product was used, followed by two to five products (n=11; 18.6%) and more than five products (n=1; 1.7%).

The main agents used in suicide attempts were prescription drugs (74.6%; n=44) (Table 1). Drugs that act on the central nervous system (CNS) were the most frequent (70.4%; n=31), with emphasis on carbamazepine (13.6%; n=8), phenobarbital (10.2%; n=6) and clonazepam (8.5%; n=5). Regarding pesticides, among the ten children who used this product, six used acetylcholinesterase inhibitors, with *chumbinho* (pellet) (n=3) being the most frequent.

**Table 1:** Variables related to suicide attempts by children seeking care at the Poison Control Center (n=59), April 1985 to December 2018.

	n	%
Time to initial care		
≤1 hour	16	27.1
1 a ≤2 hours	4	6.8
2 a ≤24 hours	30	50.8
>24 hours	2	3.4
Unknown	7	11.9
Intoxicant		
Medicines	44	74.6
Pesticides	10	16.9
Household cleaning products	3	5.1
Caustics	1	1.7
Rodenticides	1	1.7
Clinical manifestations*		
Neurological/psychic/muscular	36	61.0
Digestive	21	35.6
Ocular	7	11.9
Cardiological	6	10.2
Dermatological	3	5.1
Anatomical/functional/syndromic diagnoses	3	5.1

*Number greater than 59, since the same patient can present more than one clinical manifestation.

Gastric decontamination measures were performed in 45.8% of cases (n=27). Of these measures, there was a higher frequency of association of gastric lavage with activated charcoal (23.7%; n=14). As for the use of antagonists/antidotes, there was only a need to use atropine (5.1%; n=3).

There was a predominance of neurological/psychic/muscular manifestations (61.0%; n=36), especially drowsiness (38.9%; n=23), and digestive manifestations (35.6%; n=21), mainly vomiting (28.8%, n=17) (Table 1). Only 11.9% (n=7) of the children who received care remained asymptomatic throughout the observation period.

Most of the reasons and/or factors reported for suicide attempts were unknown or unreported (47.5%; n=28), followed by conflicts with family and/or friends (27.1%; n=16) and psychiatric disorders (15.2%; n=9) (Table 2).

**Table 2:** Reasons and/or risk factors related to children’s suicide attempts, Poison Control Center - Londrina (n=59), April 1985 to December 2018.

Reasons and/or factors related to the suicide attempt	n	%
Unknown/not informed	28	47.5
Conflicts with family/friends	16	27.1
Psychiatric disorders	9	15.2
Sexual abuse	3	5.1
Bullying	2	3.4
Other reasons	3	5.1

## DISCUSSION

In this study, it was found that suicide attempts in children were more frequent in females, with the use of a single agent, mainly from the class of drugs that act in the CNS. Neurological symptoms were the most common, especially drowsiness. As the main declared reason related to suicidal events, conflicts with family members and/or friends were identified, followed by cases related to children diagnosed with psychiatric disorders. Most patients stayed for a few days and none of them died.

There are limitations regarding data collection, since it occurred in secondary data sources. Another factor that should be highlighted is the existence of a possible underreporting regarding suicide attempts, especially in childhood. Furthermore, CIATox only reports cases of attempted suicide with the ingestion of products, excluding other causes, for example, trauma. Despite the limitations, the study is strengthened by comprehending a period of 33 years, corresponding to the entire existence of CIATox-Londrina, allowing the analysis of toxicological events involving all children, which contributes to characterize this circumstance in the child age group, and fosters new studies.

The greater risk of suicidal behavior in females has been shown in other studies.^14^ ^,^ ^15^ ^,^ ^16^ ^,^ ^17^However, despite the fact that female suicide attempts are more frequent, the effectiveness of this attempt in males is prevalent.^15^ ^,^ ^16^Bergnegger et al., described, in 2015, the contribution of the alexithymic characteristic of men to request help in depressive conditions, which makes it difficult for health services to diagnose suicide attempts.^15^

Prescription drugs were the main agents used in suicide attempts, especially those which act on the CNS, which is in line with similar studies, although with other populations.^18^ ^,^ ^19^ ^,^ ^20^The use of drugs in suicidal acts is also more prevalent in women.^21^ ^,^ ^22^

The use of gastric lavage as a measure of gastric decontamination, although commonly used, as perceived in the present study, presents itself as a controversial method. Gastric lavage may not offer benefits, even when performed up to one hour after ingestion, and still cause damage to the patient. Therefore, the recommendation for its use is rare, being a measure that should not be used routinely, indicating that consideration should be given to the use of activated charcoal, symptomatic/supportive treatment and observation of the patient instead of gastric lavage.^23^

Several studies have linked the incidence of psychiatric disorders to suicide attempts in childhood.^24^ ^,^ ^11^Psychiatric disorders in childhood are difficult to diagnose, which makes it commonly doubtful for health professionals and/or family members.^25^Child abuse (psychological, physical or sexual abuse, psychological or physical neglect) was related by Liu et al. to predisposition to suicidal behavior. Among these, emotional abuse was the most strongly related.^14^The relationship between sexual abuse and suicide attempts was confirmed by Martin et al., in 2016, who demonstrated that four out of five individuals who attempted suicide reported having suffered abuse in childhood.^26^This same study also suggests that children who suffer sexual violence are more likely to have suicidal behavior compared to those who suffer physical violence or who witness violence.^26^Traumatic childhood experiences have been associated with an increased risk of suicide in adult patients with depression. In contrast, Erol, Ersoy and Mete (2013) suggest that the occurrence of trauma in childhood is more associated with suicidal behavior than factors related to depression.^27^

In this context, health professionals must be able to care for the suicide attempt, whose occurrence requires clinical and psychological support,^3^ ^,^ ^11^and it is also necessary to know how to carefully assess the need for referral to a mental health service.^28^However, there is a difficulty for health professionals regarding the approach to patients who attempted suicide and/or their own family members to clarify information regarding the reasons and related factors, which hinders the reliability of the information.^29^

Scientific data on children with suicide attempts is scarce. According to the Notifiable Diseases Information System (SINAN) (period from 2011 to 2015), regarding notifications of self-harm and attempted suicide, there is a predominance of the adolescence (10‒19 years) and young adults (20‒39 years) age groups.^30^Thus, it is necessary to delve deeper in this important issue for public health.

Strategies to prevent these events must be adopted, such as restricting access to medicines and pesticides, the implementation of support and care programs for children and their families, early identification and adequate management of people at risk of suicide, with effective actions to reduce suicides, including the qualification of the health service and the presence of professionals to care for and monitor children, in addition to promoting sexual education in childhood, among others.^7^Therefore, scientific research should be carried out in this population, seeking to subsidise care actions and the elaboration of public policies.
